# Sex‐Specific Impact of Metabolic Dysfunction‐Associated Fatty Liver Disease on Incident Cardiovascular Diseases and Mortality

**DOI:** 10.1002/edm2.70035

**Published:** 2025-03-26

**Authors:** Mahsa Abbaszadeh, Farhad Hosseinpanah, Maryam Tohidi, Sahar Karimpour Reyhan, Maryam Mahdavi, Majid Valizadeh

**Affiliations:** ^1^ Endocrinology and Metabolism Research Center Imam Khomeini Hospital Complex, Tehran University of Medical Sciences Tehran Iran; ^2^ Obesity Research Center Research Institute for Endocrine Sciences, Shahid Beheshti University of Medical Science Tehran Iran; ^3^ Prevention of Metabolic Disorders Research Center Research Institute for Endocrine Sciences, Shahid Beheshti University of Medical Sciences Tehran Iran

**Keywords:** cardiovascular disease, metabolic dysfunction‐associated fatty liver diseases, mortality, non‐alcoholic fatty liver disease

## Abstract

**Background and Aims:**

Considering recent revisions in the nomenclature for fatty liver disease, alongside limited data on sex‐specific differences in its cardiovascular/mortality outcomes, this study aims to investigate the prevalence and impact of metabolic‐associated fatty liver disease (MAFLD) on cardiovascular disease (CVD) and mortality in men and women over a 12‐year follow‐up period.

**Methods:**

In this large population‐based cohort study, 7101 individuals aged ≥ 30 were enrolled. The prevalence of MAFLD was investigated in both genders. After excluding individuals with a history of previous CVD, 6331 participants were followed up for CVD and mortality over 12 years. Steatosis was defined as fatty liver index (FLI) ≥ 60. Multivariate‐adjusted hazard ratios (HRs) were calculated for CVD and mortality.

**Results:**

The prevalence of MAFLD was 43.2%, higher in men (46.5%) than women (40.6%). Men with MAFLD (47.7 ± 12.1) were younger than women (52.2 ± 11.1). In the 12‐year follow‐up of 6331 individuals, multivariable‐adjusted CVD HRs for MAFLD were 1.36 (1.10–1.67) in men and 1.48 (1.16–1.88) in women. Adjusted mortality HRs were 1.17 (0.86–1.59) and 1.38 (1.00–1.91) in men and women, respectively. Among patients with MAFLD, a subgroup with diabetes faced the highest hazard for CVD and mortality.

**Conclusion:**

This study found that MAFLD is more common in men at a younger age. Despite the higher prevalence in men, women with MAFLD face a greater risk of cardiovascular events and mortality. Findings highlight the importance of gender‐specific considerations in primary prevention programmes for MAFLD‐related cardiovascular disease and mortality.

## Graphical abstract

Metabolic dysfunction‐associated fatty liver disease (MAFLD) is more common in men. MAFLD raises the risk of cardiovascular events and mortality. Women with MAFLD face a greater risk of cardiovascular events and mortality. Adding steatosis to metabolic dysfunction raised cardiovascular and mortality risk.

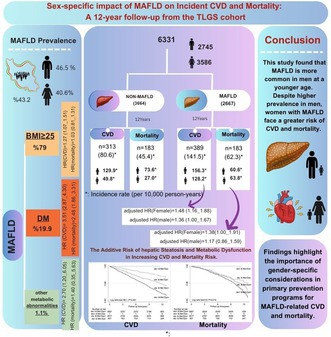

**Summary**

- Metabolic Dysfunction‐Associated Fatty Liver Disease (MAFLD) is more common in men at a younger age.
- Despite higher prevalence in men, women with MAFLD face a greater risk of Cardiovascular disease and mortality.
- This study underscores the need for gender‐specific strategies to prevent and treat these risks.

## 1 Introduction

Fatty liver disease (FLD), especially its metabolic‐associated form, has become a growing global health concern. Despite its high prevalence and severe cardiovascular, hepatic and oncologic consequences, only one newly approved treatment is available [1, 2]. Recent years have seen increased attention to this condition, leading to changes in its nomenclature, including terms like metabolic dysfunction‐associated fatty liver disease (MAFLD) [3] and metabolic dysfunction‐associated steatotic liver disease (MASLD) [4], emphasising the importance of addressing cardiometabolic disturbances alongside hepatic steatosis. MAFLD, introduced in 2020, affects approximately 38% of the global population [5] and is defined by the presence of hepatic steatosis along with one of these three additional metabolic disorders: overweight/obesity, type 2 diabetes or one of the other defined metabolic abnormalities [3].

Numerous studies have been published in recent years on the prevalence and outcomes of MAFLD in the United States, Europe and East Asia. Meanwhile, limited data are available on its prevalence and outcomes in Western Asian countries. Although studies primarily associated non‐alcoholic fatty liver disease (NAFLD) with cardiovascular events and mortality [6], there are scarce data about the outcomes of MAFLD as a newly described term. Furthermore, it is necessary to address whether the risk of cardiovascular events in MAFLD is solely associated with the combination of metabolic disorders or if the presence of steatosis independently affects and, to what extent, contributes to these outcomes.

Recent studies have examined how gender influences the onset and outcomes of FLD [7, 8]. Meanwhile, our understanding of sex differences in fatty liver is still incomplete [7]. Recognising the impact of gender on FLD is crucial for planning effective sex‐specific strategies to prevent and manage cardiovascular and mortality risks associated with the condition. This study aimed to evaluate the prevalence of MAFLD and its impact on cardiovascular disease (CVD) and mortality in men and women over a 12‐year follow‐up period within the Tehran Lipid and Glucose Study (TLGS). We also investigated whether hepatic steatosis, independent of metabolic disorders, increased the risk of these outcomes.

## 2 Methods

### 2.1 Study Population

The present study was part of the TLGS, the first large‐scale community‐based cohort study in Iran with long‐term follow‐up. TLGS aims to determine the prevalence, incidence and risk factors of non‐communicable diseases and assess the impact of a healthy lifestyle on improving these risks in a representative sample of the Tehran population, the most populated city in Iran. The TLGS study began in 1999 with 15,005 individuals aged 3 years and older, selected through random multi‐stage cluster sampling. Follow‐up data have been collected every 3 years across six examinations. The study design and details have been previously reported [9].

**FIGURE 1:**
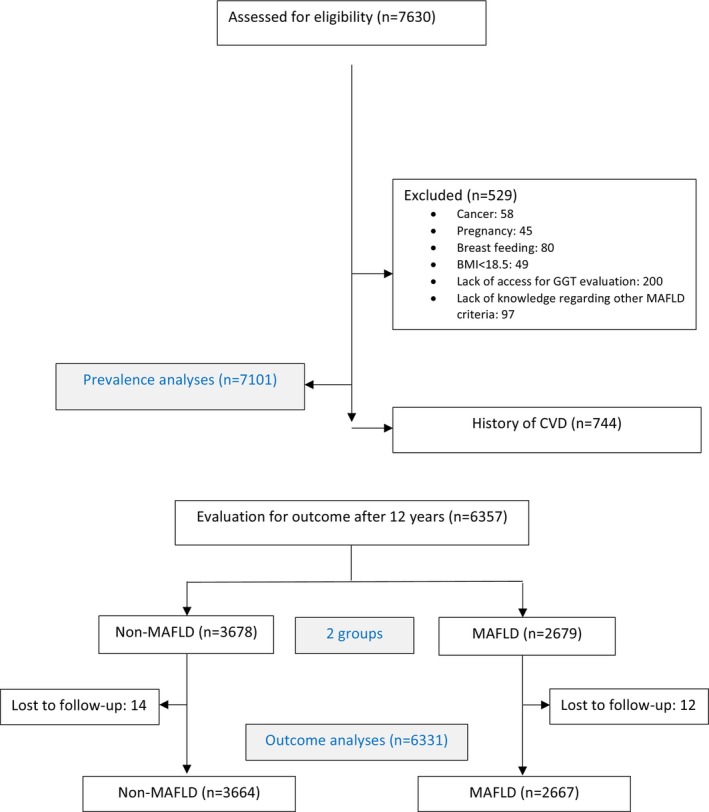
Participant flowchart. BMI, body mass index, CVD, cardiovascular death, GGT, gamma‐glutamyl transferase, MAFLD, metabolic dysfunction‐associated fatty liver disease.

In this study, all men and women aged 30 years and older from phase 3 of the TLGS have been enrolled (7630 individuals since 2006). The exclusion criteria of the study included pregnancy, lactation, history of malignancy, body mass index (BMI) less than 18.5 kg/m^2^, absence of serum samples for GGT measurement or lack of information on other variables related to the definition of MAFLD (529 individuals), and the disease prevalence was assessed for 7101 individuals. In the next stage, individuals with a history of cardiovascular disease at baseline were excluded (744 individuals), 26 were lost to follow‐up, and the final longitudinal analysis for 12‐year outcomes was performed on 6331 individuals (Figure 1).

### 2.2 Clinical and Laboratory Measurements

Standard‐validated questionnaires were employed to gather comprehensive data on the subjects' demographics, education, smoking, medication use, and medical and family history. These questionnaires were administered by trained general physicians through interviews with the participants at the beginning and during the follow‐up phases. Physical activity was determined using the Persian version of modifiable activity questionnaire (MAQ), validated in previous studies, and physical activity level was estimated in metabolic hours per week [10, 11]. Anthropometric measurements, including height, weight and waist circumference, followed the standard TLGS protocol [12, 13]. Weight was measured with minimal clothing using a calibrated digital scale (Seca707, 0.1–150 kg; Hanover, MD, USA) with 100 g accuracy, while height was recorded in an upright position, shoulders relaxed and without shoes. Waist circumference was measured at the umbilicus level based on WHO criteria. Further details regarding the assessment of family history of premature cardiovascular disease, BMI and blood pressure have been documented in previous studies [14, 15]. The tools and methods used for clinical measurements remained consistent throughout the study.

Blood samples were collected from the study participants between 7 AM and 9 AM for laboratory investigations after a fasting period of 12–14 h. Within 30–45 min after collection, the samples were centrifuged. The measurement techniques for blood glucose, total cholesterol (TC) concentration, serum triglycerides (TG), high‐density lipoprotein cholesterol (HDL‐C) concentration and creatinine have been explained in previous studies [16, 17, 18]. When serum triglycerides were below 4.52 mmol/L (400 mg per deciliter). The Friedewald formula was employed to determine the concentration of low‐density lipoprotein cholesterol (LDL‐C) [19]. To determine GGT levels, serum samples from the TLGS cohort, frozen at −80°C since 2006, were thawed. GGT concentration was determined using the enzymatic colorimetric method with the Audit kit (Delta Darman Pars Company, Tehran, Iran) using a Pictus 700 analyser (Diatron, Hungary). Commercial serum controls in normal and pathological concentration ranges were used to monitor the assay quality, and the intra‐ and inter‐assay coefficients of variation in GGT measurements were less than 2.7% and 3.6%, respectively. The reference ranges for GGT were set as 7–32 U/L for women and 11–50 U/L for men.

### 2.3 Definition of Variables

MAFLD, based on the 2020 consensus, is defined by the presence of hepatic steatosis along with at least one of the following criteria: Overweight or obesity (BMI ≥ 25 kg/m^2^), type 2 diabetes or at least two metabolic disturbances (waist circumference [WC], blood pressure, triglycerides and HDL‐C, prediabetes) [3]. This study used the fatty liver index (FLI) to define hepatic steatosis. The calculation of FLI is based on BMI, WC, TG and GGT, and its validation for the assessment of steatosis was first confirmed in Italy in 2006 [20]. It is currently accepted as a reliable indicator for defining steatosis in extensive epidemiological studies, endorsed by many clinical guidelines [21]. In this study, a fatty liver index ≥ 60 indicated steatosis. According to national studies, a waist circumference ≥ 90 cm was used as the threshold for defining high WC in both genders [22].

Prediabetes was defined as fasting plasma glucose (FPG) levels between 5.5 and 7 mmol/L or 2‐h plasma glucose between 7.7 and 11.1 mmol/L. Type 2 diabetes was defined as FPG ≥ 7 mmol/L or 2hPG ≥ 11.1 mmol/L or current use of antidiabetic medications. Hypertension was characterised by a systolic blood pressure (SBP) of 140 mmHg or higher, a diastolic blood pressure (DBP) of 90 mmHg or higher, or antihypertensive medications. Education level was grouped as < 12 or ≥ 12 years.

Physical activity, assessed using the MAQ questionnaire and categorised according to the 2005 International Physical Activity Questionnaire (IPAQ) guidelines, falls into three groups: (1) strenuous activity, defined as more than 1500 MET(metabolic equivalent task)‐minutes per week; (2) moderate activity, ranging from 600 to 1500 MET‐minutes per week; and (3) low physical activity, characterised by achieving less than 600 MET‐minutes per week [23]. This study defined ideal physical activity as achieving more than 600 MET‐minutes per week, corresponding to a level above low physical activity. Smoking was categorised as current/occasional cigarette/water pipe/pipe use (smokers) or never smoked. Having a family history of diabetes means that at least one parent or sibling has been diagnosed with the condition. The estimated glomerular filtration rate (eGFR) was determined using CKD‐EPI equations based on serum creatinine levels [24]. Chronic kidney disease (CKD) was identified as an eGFR less than 60 mL/min/1.73 m^2^.

### 2.4 Definition of Outcomes

Continuous monitoring of medical outcomes was conducted annually through phone calls administered by trained nurses. In cases where hospitalisation or mortality was related to a medical condition, a qualified physician collected supplementary information during home visits and by reviewing the hospital records [16]. All the gathered data underwent a rigorous assessment by an outcome committee formed by a team of medical specialists, including internists, endocrinologists, cardiologists, epidemiologists, pathologists and other relevant experts when necessary. This outcome committee remained unaware of the status of the baseline risk factors throughout the evaluation process.

CVD was defined to include coronary heart disease (CHD) as well as both fatal and non‐fatal stroke cases. CHD included definitive myocardial infarction (MI) diagnosed via electrocardiogram (ECG) and biomarkers, along with probable MI, characterised by significant ECG findings with cardiac symptoms and missing biomarker data or ambiguous biomarkers. Also, unstable angina pectoris involving new cardiac symptoms or alterations in symptom patterns, positive ECG findings and average biomarker values were considered. Cases confirmed through angiography were also classified as CHD. Stroke was identified as a new onset of neurological symptoms lasting more than 24 h. Confirmation of deaths due to CHD or stroke was performed by checking the death certificates or medical records.

### 2.5 Statistical Analysis

The normality of the data was evaluated using the Kolmogorov–Smirnov test and by analysing the histograms of the data. The study findings were reported as means with standard deviations or medians with interquartile ranges for quantitative variables and as frequencies with percentages for categorical variables. Independent *t*‐tests were employed to evaluate the differences in means among the groups for quantitative variables that follow a normal distribution. The Mann–Whitney test was used for quantitative variables that were not normally distributed. The chi‐squared test was utilised to assess the differences in categorical variables.

This study examined the association between MAFLD and the risk of cardiovascular events or mortality using the Multivariate Cox proportional hazard model, and the hazard ratio and 95% confidence interval were reported. The interaction effect between gender and MAFLD on the risk of cardiovascular events or mortality was examined (both *p*‐values for interaction < 0.001), and given the confirmation of this interaction, separate analyses were conducted for females and males. The connection between MAFLD and the risk of outcomes was further adjusted for potential confounding factors, including age, education level, physical activity, chronic kidney disease, family history of premature cardiovascular disease, smoking and serum cholesterol levels in the models. Diabetes and BMI, part of the main definitions of MAFLD, were not included in the models.

In this study, to evaluate whether steatosis or metabolic disorders act as independent predictors of cardiovascular events and Mortality, the participants were stratified into four subgroups: (1) no liver steatosis and no metabolic dysfunction, (2) metabolic dysfunction without liver steatosis, (3) liver steatosis in the absence of metabolic dysfunction, and (4) liver steatosis accompanied by metabolic dysfunction. The Cox proportional hazard regression test was employed, and the results were reported as hazard ratios and their 95% confidence intervals. SPSS version 26 was used to analyse data, and a *p*‐value of less than 0.05 was used as the threshold for statistical significance.

## 3 Results

In the analysis of 7101 individuals, the prevalence of MAFLD in the community was 43.2% (95% CI: 42.1%–44.4%) and higher in men (46.5%, 95% CI: 44.7%–48.2%) than in women (40.6%, 95% CI: 39.1%–42.1%). Of the 6331 individuals in a 12‐year follow‐up study, 56.6% were female (*n* = 3586). The women's average age was 48.0 ± 11.8 years, and the men's 48.8 ± 13.1 years. The age of women with MAFLD (52.2 ± 11.1) was notably higher than that of men with MAFLD (47.7 ± 12.1). Table 1 outlines the baseline characteristics of the participants, classified by gender and MAFLD status (Table 1).

**TABLE 1:** Baseline characteristics of the study participant by MAFLD status: Tehran Lipid and Glucose Study.

Characteristics	Women (3586)				Men (2745)				*p*
	Total	Non‐MAFLD	MAFLD	*p*	Total	Non‐MAFLD	MAFLD	*p*	
Age (years)	48.0 ± 11.8	45.4 ± 11.4	52.2 ± 11.1	< 0.001	48.8 ± 13.1	49.8 ± 13.1	47.7 ± 12.1	< 0.001	0.012
Height (cm)	155.6 ± 6.0	156.4 ± 6.0	154.4 ± 5.9	< 0.001	170.1 ± 6.8	169.8 ± 6.9	170.6 ± 6.6	< 0.001	< 0.001
Weight (kg)	70.5 ± 12.0	65.0 ± 8.5	79.4 ± 11.5	< 0.001	78.6 ± 13.1	71.2 ± 9.0	87.2 ± 11.7	0.001	< 0.001
BMI (kg/m^2^)	29.2 ± 4.8	26.6 ± 3.1	33.3 ± 4.1	< 0.001	27.1 ± 4.0	24.7 ± 2.5	29.9 ± 3.4	< 0.001	< 0.001
WC (cm)	91.5 ± 12.5	84.4 ± 8.8	102.6 ± 8.6	< 0.001	96.4 ± 10.0	90.2 ± 7.3	103.4 ± 7.7	< 0.001	< 0.001
TC (mg/dL)	199.2 ± 39.4	190.2 ± 36.2	213.5 ± 40.1	< 0.001	192.4 ± 37.5	185.2 ± 35.1	200.7 ± 38.4	< 0.001	< 0.001
TG (mg/dL)	135 (96–193)	110 (81–148)	188 (141–251)	< 0.001	151 (106–215)	120 (89–160)	200 (148–275)	< 0.001	< 0.001
HDL‐C (mg/dL)	44.3 ± 10.3	45.9 ± 10.3	41.8 ± 9.6	< 0.001	37.5 ± 8.5	39.5 ± 8.9	35.2 ± 7.37	< 0.001	< 0.001
LDL‐C (mg/dL)	123.8 ± 33.0	120.0 ± 32.0	130.3 ± 33.8	< 0.001	120.9 ± 32.3	119.5 ± 31.2	122.5 ± 33.7	0.02	< 0.001
GGT (U/L) (median IQR)	17 (13–23)	14 (12–18)	22 (17–33.5)	< 0.001	25 (19–35)	21 (16–27)	32 (24–44)	< 0.001	< 0.001
FLI	48.5 ± 29.2	28.5 ± 16.9	80.1 ± 11.1	< 0.001	55.2 ± 26.1	34.6 ± 16.1	78.9 ± 10.8	< 0.001	< 0.001
FPG	98.8 ± 32.8	92.5 ± 25.8	108.8 ± 39.6	< 0.001	98.4 ± 29.2	95.0 ± 27.2	102.3 ± 30.9	< 0.001	0.59
eGFR (mL/min/1.73 m^2^)	70.9 ± 13.7	72.6 ± 13.5	68.4 ± 13.8	< 0.001	75.4 ± 13.4	75.0 ± 13.4	75.8 ± 13.4	0.129	< 0.001
SBP (mm Hg)	114.8 ± 19.4	109.9 ± 17.5	122.6 ± 19.8	< 0.001	119.3 ± 17.7	116.8 ± 17.7	122.2 ± 17.3	< 0.001	< 0.001
DBP (mm Hg)	73.4 ± 10.16	71.1 ± 9.5	77.1 ± 10.0	< 0.001	76.7 ± 10.2	74.5 ± 9.9	79.3 ± 9.9	< 0.001	< 0.001
Diabetes mellitus	13.5%	7.3%	23.5%	< 0.001	11.3%	7.1%	16.1%	< 0.001	0.008
BMI ≥ 25	81.1%	69.4%	99.6%	< 0.001	70.0%	46.5%	97.0%	< 0.001	< 0.001
Obesity (BMI ≥ 30)	38.3%	12.9%	78.4%	< 0.001	20.9%	1.1%	43.6%	< 0.001	< 0.001
FH of premature CVD	11.0%	10.7%	11.4%	0.512	8.7%	7.5%	10.2%	0.015	0.003
Hypertension	17.8%	11.0%	28.7%	< 0.001	18.6%	15.7%	22.0%	< 0.001	0.426
Dyslipidaemia	89.1%	84.1%	97.0%	< 0.001	85.7%	78.0%	94.5%	< 0.001	< 0.001
Chronic kidney disease	20.4%	16.8%	25.9%	< 0.001	12.2%	13.2%	11.2%	0.13	< 0.001
Smoker	2.9%	2.9%	2.8%	0.91	24.6%	24.9%	24.2%	0.69	< 0.001
Ideal physical activity (min/week)	31.0%	28.8%	34.6%	< 0.001	40.7%	38.7%	42.9%	0.03	< 0.001
Education ≥ 12 years	12.0%	16.0%	5.5%	< 0.001	23.3%	24.0%	22.4%	0.32	< 0.001

*Note:* Data are the number (%), mean ± SD, or median (interquartile range). Hepatic steatosis was identified using a fatty liver index of 60 or higher to diagnose MAFLD. Dyslipidaemia: Chol ≥ 200 or TG ≥ 150 or LDL ≥ 130.

Abbreviations: BMI, body mass index; DBP, diastolic blood pressure; eGFR, estimated glomerular filtration rate‐EPI; FH, family history; CVD, cardiovascular disease; FLI, fatty liver index; FPG, fasting plasma glucose; GGT, gamma‐glutamyl transpeptidase; HDL‐C, high‐density lipoprotein cholesterol; LDL‐C, low‐density lipoprotein cholesterol; MAFLD, metabolic dysfunction‐associated fatty liver disease; P‐V, *p*‐value; SBP, systolic blood pressure; TC, total cholesterol; TG, triglyceride; WC, waist circumference.

**FIGURE 2:**
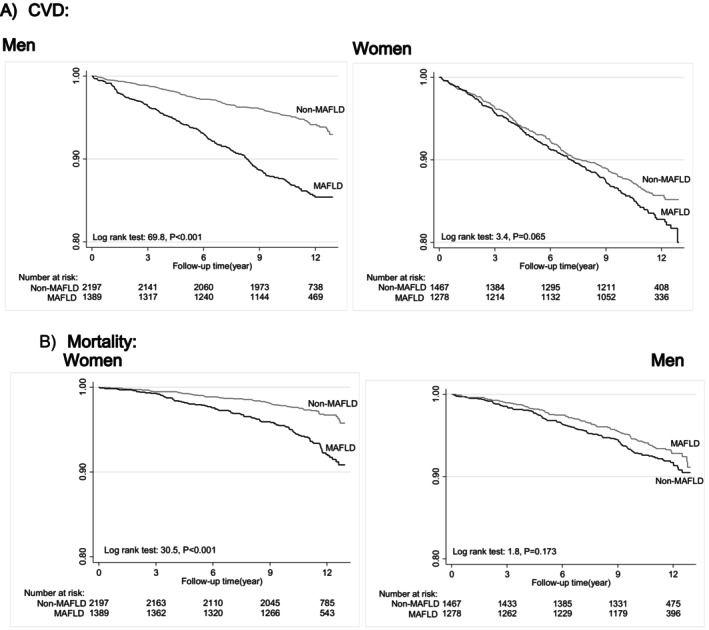
Kaplan–Meier curves for the risk of cardiovascular disease and mortality in MAFLD for both genders. MAFLD (continuous line), those without MAFLD (grey line). CI, confidence interval; CVD, cardiovascular disease; HR, hazard ratio; MAFLD, metabolic dysfunction‐associated fatty liver disease.

In this study, 702 new CVD events and 366 mortality cases occurred during 12 years of follow‐up. Incidence rate (per 10,000 person‐years) for CVD and mortality in patients with MAFLD was 141.5 (128.2–156.3) and 62.3 (53.8–71.9), respectively. Figure 2 illustrates Kaplan–Meier curves for cumulative survival free from CVD and mortality in MAFLD for both genders. The survival curves in the women differed significantly in the MAFLD and non‐MAFLD groups (log‐rank test, *p* < 0.001) (Figure 2).

HRs for incident CVD and mortality in MAFLD and non‐MAFLD groups among 6331 study participants are shown in Table 2. An unadjusted model showed that MAFLD significantly elevated the risk of CVD and mortality in the population. This association remained unchanged even after adjusting for age, sex, education level, physical activity, chronic kidney disease, family history of premature CHD, smoking and serum total cholesterol levels [HR for CVD: 1.46 (1.25–1.71) and HR for mortality: 1.28 (1.03–1.60), *p* < 0.05]. In all models, the risk of CVD and mortality was higher in women than in men (Table 2).

**TABLE 2:** Hazard ratio (95% CI) for incident cardiovascular disease and mortality in 12 years of follow‐up based on MAFLD status.

	CVD		Mortality	
	Non‐MAFLD	MAFLD	Non‐MAFLD	MAFLD
Total (6331)				
Number of person‐years	38,808.8	27,481.3	40,331.1	29,385.54
Number of incidences	313	389	183	183
Incidence rate (per 10,000 person‐years), (95% CI)	80.6 (72.2–90.1)	141.5 (128.2–156.3)	45.4 (39.2–52.4)	62.3 (53.8–71.9)
Hazard ratio (95% CI)				
Unadjusted model	1 (reference)	1.75 (1.51–2.04)^a^	1 (reference)	1.37 (1.12–1.68)^a^
Model 1	1 (reference)	1.61 (1.38–1.86)^a^	1 (reference)	1.32 (1.07–1.63)^a^
Model 2	1 (reference)	1.63 (1.40–1.90)^a^	1 (reference)	1.30 (1.04–1.60)^a^
Model 3	1 (reference)	1.62 (1.39–1.88)^a^	1 (reference)	1.28 (1.03–1.60)^a^
Model 4	1 (reference)	1.46 (1.25–1.71)^a^	1 (reference)	1.28 (1.03–1.60)^a^
Female (*n* = 3586)				
Number of person‐years	23880.9	14430.7	24441.8	15358.7
Number of incidences	119	185	66	98
Incidence rate per 10,000 person‐years (95% CI)	49.8 (41.6–59.6)	128.2 (111.0–148.1)	27.0 (21.2–34.4)	63.8 (52.3–77.7)
Hazard ratio (95% CI)				
Unadjusted model	1 (reference)	2.57 (2.05–3.24)^a^	1 (reference)	2.35 (1.72–3.20) ^⁎^
Model 1#	1 (reference)	1.67 (1.32–2.11)^a^	1 (reference)	1.42 (1.04–1.94) ^⁎^
Model 2	1 (reference)	1.62 (1.28–2.05)^a^	1 (reference)	1.33 (0.97–1.84)
Model 3	1 (reference)	1.61 (1.27–2.40)^a^	1 (reference)	1.32 (0.96–1.82)
Model 4	1 (reference)	1.48 (1.16–1.88)^a^	1 (reference)	1.38 (1.00–1.91)
Male (*n* = 2745)				
Number of person‐years	14,927.8	1,3050.7	15889.3	14026.8
Number of incidences	194	204	117	85
Incidence rate per 10,000 person‐years (95% CI)	129.9 (112.9–149.6)	156.3 (136.3–179.3)	73.6 (61.4–88.3)	60.6 (49.0–74.9)
Hazard ratio (95% CI)				
Unadjusted model	1 (reference)	1.20 (0.99–1.46)	1 (reference)	0.82 (0.62–1.09)
Model 1#	1 (reference)	1.42 (1.16–1.73)^a^	1 (reference)	1.17 (0.89–1.56)
Model 2	1 (reference)	1.48 (1.21–1.82)^a^	1 (reference)	1.17 (0.87–1.58)
Model 3	1 (reference)	1.48 (1.20–1.81)^a^	1 (reference)	1.17 (0.87–1.58)
Model 4	1 (reference)	1.36 (1.10–1.67)^a^	1 (reference)	1.17 (0.86–1.59)

*Note:* Total number of non‐MAFLD: 3664, Total number of MAFLD: 2667. Model 1: adjusted for age and sex. Model 1#: adjusted for age. Model 2: adjusted for education and physical activity in addition to model 1. Model 3: adjusted for chronic kidney disease and family history of premature CVD in addition to model 2. Model 4: adjusted for smoking status and cholesterol in addition to model 3.

Abbreviations: CI, confidence interval; CVD, cardiovascular disease; MAFLD, metabolic dysfunction‐associated fatty liver disease.

^a^ Compared to Non‐MAFLD group as reference, *p*‐value < 0.05.

**FIGURE 3:**
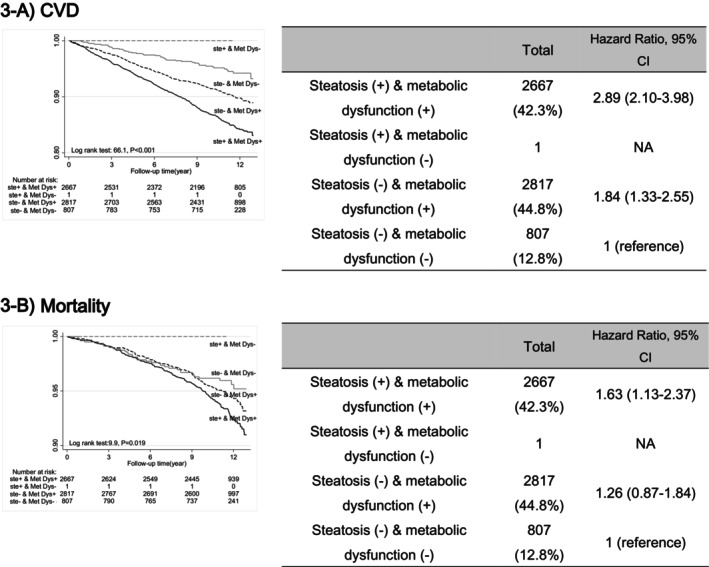
CVD events and mortality in MAFLD by the existence of either hepatic steatosis and/or metabolic dysfunction for 12 years of follow‐up. (1) No hepatic steatosis and no metabolic dysfunction (normal control; dashed grey line), (2) no hepatic steatosis but metabolic dysfunction (solid grey line), (3) hepatic steatosis without metabolic dysfunction (dashed black line), (4) hepatic steatosis with metabolic dysfunction (MAFLD; solid black line). NA, not applicable.

The study results showed that metabolic dysfunction independently elevated the risk of CVD in individuals without liver steatosis (HR: 1.84, CI 1.33–2.55 for CVD). The addition of liver steatosis to metabolic abnormalities (i.e. MAFLD) significantly increased the risk of CVD and mortality (HR: 2.89, 95% CI: 2.10–3.98, *p* < 0.001 for CVD and HR: 1.63, 95% CI: 1.13–2.37, *p* < 0.021 for mortality) (Figure 3). Among the three subgroups of MAFLD, that is, (1) steatosis with BMI ≥ 25 (*n* = 2028, 79%), (2) steatosis with diabetes (*n* = 512, 19.9%) and (3) steatosis with other metabolic disorders except for diabetes and overweight/obesity (*n* = 29, 1.1%), the highest risk of CVD was observed in the group with diabetes (HR: 3.51, 95% CI: 2.87–4.30, *p* < 0.001), and the lowest risk was in the group with overweight/obesity (HR:1.27, 95% CI (1.07–1.51), *p* = 0.007).

## 4 Discussion

In this large population‐based prospective cohort in the MENA region, we examined the prevalence of MAFLD and the incidence of CVD and mortality in both genders during a 12‐year follow‐up in Iran. The prevalence of MAFLD was 43.2%, with higher rates observed in men than women. A 12‐year follow‐up revealed higher CVD and mortality risk in patients with MAFLD, especially among women (HR: 1.48 for CVD and HR: 1.38 for mortality) compared to men (HR: 1.36 for CVD and HR: 1.17 for mortality). Adding steatosis to metabolic dysfunction significantly raised CVD and mortality risk (HR: 2.89 compared to 1.84); this finding demonstrates the independent role of steatosis in increasing CVD and mortality risk.

The prevalence of MAFLD is higher in the Iranian population compared to global statistics. According to a 2022 meta‐analysis by Chan et al., the prevalence of MAFLD was reported to be 38.7% [5]. In the Iranian community, the consumption of high glycaemic index and glycaemic load carbohydrates, mainly refined grains, significantly exceeds the global average, accounting for over 62% of daily energy intake [25]. This dietary pattern may contribute to insulin resistance, increased body fat stores and an unfavourable lipid profile, potentially exacerbating metabolic dysfunction [26]. Additionally, the recent urbanisation trend in Iranian society, characterised by reduced physical activity and decreased consumption of vegetables and seafood, has been implicated in the prevalence of MAFLD [27].

This study found that MAFLD raised the risk of cardiovascular events by 46% and mortality by 28% in the total population after adjusting for confounding factors. A systematic review by Quek et al., involving over 12 million individuals, revealed a 49% increased risk of cardiovascular events associated with MAFLD [28]. While the independent role of fatty liver in CVD remains uncertain, several mechanisms support the association between NAFLD and CVD, including disruptions in lipoprotein metabolism, impaired endothelial function, increased atherosclerotic lesions and compromised compensatory responses [29, 30]. Recent studies highlight that MAFLD, focusing on metabolic risk factors, has a stronger association with CVD outcomes than NAFLD [31]. The shift from exclusionary criteria in NAFLD to the inclusionary approach in MAFLD allows for better identification of high‐risk individuals for cardiovascular diseases [32].

While MAFLD is more common in men and affects them at a younger age, this study revealed a greater risk in women in terms of CVD and mortality. The higher prevalence in men was in line with other research showing a higher prevalence of fatty liver in men [31, 32]. Men with MAFLD had significantly higher average Fatty Liver Index (FLI) values and associated factors such as waist circumference, triglycerides and GGT, despite women having slightly higher BMIs. Consequently, the prevalence of pure steatosis was notably higher in men. Moreover, women with MAFLD were older, while men with MAFLD were younger compared to those without MAFLD. This age difference may be attributed to the protective influence of oestrogen on metabolic disorders in premenopausal women, as oestrogen helps regulate lipid metabolism, insulin sensitivity, body composition and fat distribution. With menopause and the subsequent decline in oestrogen levels, these protective effects diminish, leading to increased central adiposity and metabolic risk factors, which elevate CVD and mortality risks [33].

Regarding the greater risk of fatty liver complications in women, prior studies mainly focused on hepatic complications; they reported similar results about its prevalence and progression, although concerning fibrosis and liver‐related outcomes in both men and women. In a 2020 meta‐analysis involving over 62,000 individuals with NAFLD, it was apparent that women exhibit a lower susceptibility to developing NAFLD compared to men; nonetheless, upon NAFLD onset, women encounter a heightened risk of advanced fibrosis in contrast to men [8]. We found the same data about CVD and mortality complications. A study by Miao et al., conducted on approximately 350,000 participants from the UK Biobank Study with a 12‐year follow‐up, examined the sex‐specific association between MASLD and CVD. The results showed that MASLD is an independent risk factor for CVD. While women with MASLD had higher rates of CVD mortality, CHD, ischemic stroke and atrial fibrillation compared to men, this difference was statistically significant only for ischemic stroke. The study suggested that sex‐specific vascular physiology, with women showing greater sensitivity to chronic metabolic exposures, might explain these differences [34]. Despite extensive research on FLD and its cardiovascular consequences, sex‐stratified clinical studies are still limited, leaving many aspects unclear.

In the overall population, men have a greater risk of CVD and mortality events [35]. However, as shown in our study and supported by other research, in the presence of MAFLD, this risk increases in women to the extent that it surpasses men's. The survey by Goossens et al. [36] has clarified how adipose tissue, muscles and the liver contribute to sexual dimorphism in cardiometabolic health. The study highlighted the role of epigenetics, genetics and sex hormones in shaping differences in fat distribution, skeletal muscle function and liver metabolism, which help explain the variations in insulin sensitivity and metabolic health between genders. Moreover, the study revealed that before menopause, women have a diminished risk of developing cardiometabolic diseases compared to men of the same age and body mass index. Premenopausal women tend to have higher fat mass for a given BMI but predominantly accumulate excess energy in their subcutaneous fat, especially in lower‐body fat deposits, compared to men. Meanwhile, this pattern changes after menopause. Regarding sex hormones in fatty liver, a meta‐analysis by Jaruvongvanich et al. demonstrated that men with NAFLD typically had lower levels of total testosterone (TT). In comparison, women with NAFLD generally had higher TT levels [37]. Both mechanisms of sexual dimorphism and hormonal changes can influence the onset of CVD in MAFLD in both genders.

This study reveals the independent role of steatosis in CV event risk. A 2020 Korean cohort study involving approximately 5000 individuals showed that irrespective of traditional metabolic risk factors like hypertension, diabetes, dyslipidaemia and obesity, fatty liver heightens the risk of thoracic aortic calcification and advanced atherosclerosis solely due to steatosis. While the exact mechanism remains unclear, the heightened production of inflammatory cytokines such as C‐reactive protein, interleukin 6 and tumour necrosis factor‐alpha from hepatic adipose tissue associated with steatosis may play a significant role in this association [38]. Furthermore, Gofton et al., in their study, referenced articles suggesting that in the context of MAFLD, metabolic dysregulation features drive mortality risk rather than the underlying MAFLD diagnosis itself [31]. Few clinical studies have been conducted in this area, and additional research is required to confirm the role of steatosis in increasing cardiovascular and mortality risks. Definitions such as MAFLD and MASLD may provide better aggregation of metabolic disorders for predicting cardiovascular events and mortality than previous metabolic syndrome definitions.

The current study has several limitations. First, we did not have access to liver histology and imaging to define steatosis, and we relied on the FLI for this purpose. Although many studies suggest that FLI, especially in epidemiological studies, is an acceptable marker for defining hepatic steatosis [3, 20, 21], and numerous recent studies have used it for assessing steatosis [39, 40], there are challenges in determining the optimal cut‐off between 30 and 60 for defining hepatic steatosis. According to the primary study, an FLI < 30 can rule out steatosis (SN = 87%; LR− = 0.2), and an FLI ≥ 60 can rule it in (SP = 86%; LR+ = 4.3). In our study, we used a cut‐off of 60 to define steatosis but also performed a sensitivity analysis with a cut‐off of 30. This analysis showed a higher prevalence of MAFLD at lower FLI levels and highlighted more distinct differences in the association between CVD, mortality and MAFLD across genders (Supplementary 1). Second, to calculate the FLI and measure GGT values, we had to thaw samples from 12 years ago. Initially, we were concerned about the samples' accuracy. Still, previous studies have confirmed the accuracy of GGT measurements from thawed serum samples taken 12 and 25 years before [41, 42]. Third, for the MAFLD diagnosis, we had no access to some metabolic variables in our samples, as none had hs‐CRP, and only half had insulin and HOMA‐IR levels. The subgroup of MAFLD with metabolic disorders (except for overweight/obesity and diabetes) represents a meagre percentage of all MAFLD in most studies (below 3%) [43], suggesting its minimal impact on the overall analysis. Although having data on NAFLD and comparing it with MAFLD could have enriched the study, cultural and legal constraints prevented us from accessing information about alcohol consumption, making it impossible to compare the prevalence and outcomes of NAFLD and MAFLD. Another limitation was the lack of access to actual LDL‐C measurements; instead, we relied on estimated LDL‐C values calculated using the Friedewald formula. This formula assumes a fixed TG: VLDL‐C ratio of 5, which can vary among individuals and under specific metabolic conditions. This variability reduces the method's accuracy, particularly in cases where LDL‐C is less than 70 mg/dL or triglycerides are ≥ 200 mg/dL [44]. Finally, we must interpret our results cautiously when extrapolating them to other populations, as they were obtained from a sample of middle‐aged Iranian adults.

The main strengths of this population‐based cohort in the MENA region lie in its prospective design, large cohort size and adherence to standardised anthropometric and laboratory data collection methods. Additionally, the study benefited from an extensive follow‐up period of 12 years. In the present research, we calculated the prevalence of MAFLD and determined the Hazard ratio for cardiovascular and mortality outcomes for the first time in the MENA region. The follow‐up was conducted very accurately, with just 26 cases of loss to follow‐up over this time. Follow‐up assessments were conducted through a meticulous outcome review committee comprising several physicians and nurses with access to individuals' medical records. Furthermore, we conducted a sex‐stratified analysis of MAFLD prevalence and outcomes, which can help develop sex‐specific strategies to prevent and treat this condition.

## 5 Conclusion

This research discovered that MAFLD is more common in men at a younger age. Over a 12‐year follow‐up in a community‐based cohort, we demonstrated that MAFLD raises the risk of cardiovascular events and mortality, especially in women. Despite higher prevalence in men, women face a greater risk of cardiovascular events and mortality. These findings highlight the importance of gender‐specific considerations in planning effective strategies for preventing and managing cardiovascular and mortality risks associated with the condition. Nevertheless, considering the dynamic nature of fatty liver and metabolic abnormalities, further longitudinal assessments could enhance understanding of the changing metabolic profile related to MAFLD. Further, longitudinal studies are thus needed to more accurately assess the sex‐specific impact of MAFLD on CVD and mortality.

## Author Contributions

Study concept and design: Majid Valizadeh and Farhad Hosseinpanah; Data gathering, analysis and interpretation of data: Mahsa Abbaszadeh, Maryam Tohidi and Maryam Mahdavi; Drafting of the manuscript: Mahsa Abbaszadeh; Critical revision of the manuscript for important intellectual content: Farhad Hosseinpanah, Sahar Karimpour Reyhan and Majid Valizadeh; statistical analysis: Maryam Mahdavi. All authors reviewed the manuscript.

## Ethics Statement

The proposed research received approval from the National Ethics Committee on Biomedical Research of Iran (Code: IR.SBMU.ENDOCRINE.REC.1401.103) on 1 February 2023, ensuring compliance with ethical standards. Additionally, each subject provided written informed consent before participation.

## Conflicts of Interest

The authors declare no conflicts of interest.

## Supporting information


Data S1.

## Data Availability

The authors own all the data and material and are available on demand.
