# Supplementary material for: Sex‐Specific Impact of Metabolic Dysfunction‐Associated Fatty Liver Disease on Incident Cardiovascular Diseases and Mortality
Source: Endocrinol Diabetes Metab. 2025 Mar 26;8(3):e70035. doi: 10.1002/edm2.70035 (PMC11946537; doi:10.1002/edm2.70035)
Supplement: Supplementary file 1 — Data S1. [file EDM2-8-e70035-s001.docx]

Hazard ratio (95% CI) for incident cardiovascular disease and mortality over 12 years of follow-up based on MAFLD status. A sensitivity analysis was conducted using a lower FLI cutoff of 30. Text presented in black refers to the analysis based on an FLI cutoff of 60, while text in red corresponds to the analysis using an FLI cutoff of 30.

|  | CVD . | | Mortality . | |
| --- | --- | --- | --- | --- |
|  | Non-MAFLD  N=3664  N=1844 | MAFLD  2667  4487 | Non-MAFLD  3664  1844 | MAFLD  2667  4487 |
| Total (6331) |  |  |  |  |
| Number of person-years | 38808.8 | 27481.3 | 40331.1 | 29385.54 |
| Number of incidences | 313 | 389 | 183 | 183 |
| Incidence rate (per 10,000 person-years), (95% CI) | 80.6 (72.2-90.1) | 141.5 (128.2-156.3) | 45.4 (39.2-52.4) | 62.3 (53.8-71.9) |
| Hazard ratio (95% CI) |  |  |  |  |
| Unadjusted model | 1 (reference) | 1.75 (1.51-2.04) ^⁎^  2.05 (1.68-2.49)^*^ | 1 (reference) | 1.37 (1.12-1.68) ^⁎^  1.32(1.04-1.68)* |
| Model 1 | 1 (reference) | 1.61 (1.38-1.86) ^⁎^  1.65 (1.36-2.02)* | 1 (reference) | 1.32 (1.07-1.63) ^⁎^  1.06 (0.83-1.35) |
| Model 2 | 1 (reference) | 1.63 (1.40-1.90) ^⁎^  1.73 (1.41-2.13)* | 1 (reference) | 1.30 (1.04-1.60) ^⁎^  1.04 (0.80-1.34) |
| Model 3 | 1 (reference) | 1.62 (1.39-1.88) ^⁎^  1.72 (1.40-2.12)* | 1 (reference) | 1.28 (1.03-1.60) ^⁎^  1.04 (0.80-1.34) |
| Model 4 | 1 (reference) | 1.46 (1.25-1.71) ^⁎^  1.53 (1.24-1.88)^*^ | 1 (reference) | 1.28 (1.03-1.60) ^⁎^  1.04 (0.80-1.35) |
|  |  |  |  |  |
| Female (n=3586) |  |  |  |  |
| Number of person-years | 23880.9 | 14430.7 | 24441.8 | 15358.7 |
| Number of incidences | 119 | 185 | 66 | 98 |
| Incidence rate per 10,000 person-years (95% CI) | 49.8 (41.6-59.6) | 128.2 (111.0-148.1) | 27.0 (21.2-34.4) | 63.8 (52.3-77.7) |
| Hazard ratio (95% CI) |  |  |  |  |
| Unadjusted model | 1 (reference) | 2.57 (2.05-3.24) ^⁎^  3.69 (2.63-5.19)* | 1 (reference) | 2.35 (1.72-3.20) ^⁎^  2.66 (1.75-4.05)* |
| Model 1^#^ | 1 (reference) | 1.67 (1.32- 2.11) ^⁎^  1.97 (1.40-2.79)* | 1 (reference) | 1.42 (1.04-1.94) ^⁎^  1.21(0.80-1.81) |
| Model 2 | 1 (reference) | 1.62 (1.28- 2.05) ^⁎^  1.97 (1.38-2.80)* | 1 (reference) | 1.33 (0.97-1.84)  1.18(0.77-1.80) |
| Model 3 | 1 (reference) | 1.61 (1.27- 2.40) ^⁎^  1.96 (1.38-2.79)* | 1 (reference) | 1.32 (0.96-1.82)  1.17(0.76-1.80) |
| Model 4 | 1 (reference) | 1.48 (1.16-1.88) ^⁎^  1.77 (1.24-2.53)* | 1 (reference) | 1.38 (1.00-1.91)  1.23(0.80-1.91) |
|  |  |  |  |  |
| Male (n=2745) |  |  |  |  |
| Number of person-years | 14927.8 | 13050.7 | 15889.3 | 14026.8 |
| Number of incidences | 194 | 204 | 117 | 85 |
| Incidence rate per 10,000 person-years (95% CI) | 129.9 (112.9-149.6) | 156.3 (136.3-179.3) | 73.6 (61.4-88.3) | 60.6 (49.0-74.9) |
| Hazard ratio (95% CI) |  |  |  |  |
| Unadjusted model | 1 (reference) | 1.20 (0.99-1.46)  1.15(0.90-1.47) | 1 (reference) | 0.82 (0.62-1.09)  0.70 (0.51-0.93)* |
| Model 1^#^ | 1 (reference) | 1.42 (1.16-1.73) ^⁎^  1.33 (1.04-1.80)* | 1 (reference) | 1.17 (0.89-1.56)  0.92(0.68-1.25) |
| Model 2 | 1 (reference) | 1.48 (1.21-1.82) ^⁎^  1.41 (1.09-1.83)* | 1 (reference) | 1.17(0.87-1.58)  0.89(0.65-1.24) |
| Model 3 | 1 (reference) | 1.48 (1.20-1.81) ^⁎^  1.41 (1.09-1.82)* | 1 (reference) | 1.17 (0.87-1.58)  0.90(0.65-1.25) |
| Model 4 | 1 (reference) | 1.36 (1.10-1.67) ^⁎^  1.28(0.98-1.66) | 1 (reference) | 1.17 (0.86-1.59)  0.90(0.64-1.25) |

MAFLD, metabolic dysfunction-associated fatty liver disease; CVD, cardiovascular disease; CI, confidence interval.

Model 1: adjusted for age and sex.

Model 1#: adjusted for age.

Model 2: adjusted for education and physical activity in addition to model 1.

Model 3: adjusted for chronic kidney disease, and family history of premature CVD in addition to model 2.

Model 4: adjusted for smoking status and cholesterol in addition to model 3.

⁎ Compared to Non-MAFLD group as reference, P value < 0.05.
